# Odor Communication and Mate Choice in Rodents

**DOI:** 10.3390/biology7010013

**Published:** 2018-01-25

**Authors:** Michael H. Ferkin

**Affiliations:** Department of Biological Sciences, University of Memphis, Memphis, TN 38017, USA; mhferkin@memphis.edu

**Keywords:** life history strategies, mate choice, odor communication, over-marks, scent marks, rodents

## Abstract

This paper details how chemical communication is affected by ecological challenges such as finding mates. I list several conditions that affect the decision to attract mates, the decision to respond to the signals of potential mates and how the response depends on context. These mate-choice decisions and their outcomes will depend on the life history constraints placed on individuals such as their fecundity, sex, lifespan, opportunities to mate in the future and age at senescence. Consequently, the sender’s decision to scent mark or self-groom as well as the receiver’s choice of response represents a tradeoff between the current costs of the participant’s own survival and future reproduction against that of reproducing now. The decision to scent mark and the response to the scent mark of opposite-sex conspecifics should maximize the fitness of the participants in that context.

## 1. Introduction and Overview

Identification of suitable mates is probably a universal need for all species that reproduce sexually. Chemical signals often provide crucial information about the sex and reproductive condition of conspecifics. In many species of terrestrial mammals, individuals are more attracted to odors of sexually receptive, opposite-sex conspecific than those of same-sex conspecifics [1,2]. Often, males are not attracted to females that are not sexually receptive [3,4]. For instance, scent marks deposited by females during estrus or behavioral estrus are more attractive than those deposited by females outside of estrus to male conspecifics in house mice, *Mus musculus* and golden hamsters, *Mesocricetus auratus* [5,6,7,8], brown lemmings, *Lemmus trimucronatus* and collared lemmings, *Dicrostonyx groenlandicus* [9], meadow voles, *Microtus pennsylvanicus* [10] (female meadow voles are induced ovulators and during the breeding season or under long photoperiods are in behavioral estrus unless they are pregant or lactating; female voles do not have an estrous cycle and females voles in behavioral estrus are sexually receptive), Indian desert gerbils, *Meriones hurrianae* [11], Mongolian gerbils, *Meriones unguiculatus* [12], woodrats, *Neotoma lepida* [13], Columbian ground squirrels, *Spermophilus columbianus* [14], dogs, *Canis lupus familiaris* [15], rams, *Ovis aries* [16] and pygmy marmosets, *Cebuella pygmaea* [17]. Thus, females approaching or in estrus may release odors that advertise their sexual receptivity [5]. In addition, the changes in the attractiveness of a female’s scent mark appear to be concomitant with changes in their behavior. Female golden hamsters increase their vaginal and flank marking immediately prior to sexual receptivity, concentrating those scent marks around dominant male territories and also creating a trail of scent marks between the burrow of a dominant male and her own burrow [18].

In many species of mammals, females also advertise their sexual receptivity during postpartum estrus. Postpartum estrus is a relatively short period of heightened sexual receptivity that occurs immediately after parturition [19,20]. Females that mate and become pregnant during postpartum estrus gestate a set of offspring while they are nursing the previous litter. Postpartum estrus females are simultaneously pregnant and lactating, which maximizes the number of litters that they can produce during the breeding season. Postpartum estrus occurs in many species but it is especially prevalent in small-sized rodents and in lagomorphs [19,20]. Postpartum estrus female mammals produced odors and scent marks that are more attractive to males than are those produced by females not in postpartum estrus [21,22,23]. Females in postpartum estrus are also more likely to become pregnant than females not in postpartum estrus [20]. Thus, in some species like meadow voles and prairie voles, *Microtus ochrogaster*, postpartum estrus females increase scent marking, over-marking and self-grooming when they encountered the scent marks of males compared to females not in postpartum estrus [20,21].

From a physiological point of view, the attractiveness of odors of the opposite sex is affected by titers of circulating free testosterone and estradiol of both the receiver and sender of the odors. These steroid hormones were often necessary for males and females, respectively, to produce odors that were attractive to opposite-sex conspecifics [24,25]. Gonadectomy usually reduces the attractiveness of opposite-sex odors and the attractive properties of such odors can be restored by the appropriate hormone replacement therapy [24,25,26,27]. Similarly, the attractiveness of odors of opposite-sex conspecifics was also dependent on the hormonal milieu of the receiver. Typically, receivers with low circulating titers of gonadal steroids are not attracted to the odors of sexually receptive, opposite-sex conspecifics [24,25]. However, gonadectomized males and ovariectomized females that treated with replacement testosterone or estrogen, respectively, become attracted to the odors of opposite-sex conspecifics [26,28]. For many seasonally-reproducing mammals, high titers of gonadal hormones during the breeding season are necessary for senders to produce odors that are attractive to opposite-sex conspecifics and receivers to respond preferentially to them. Gonadal regression and low titers of gonadal steroids cause individuals to no longer produce odors that are attractive to the opposite sex reproductive, which corresponds with reproductive quiescence [24,25,26,27,28].

The influence of an individual’s hormonal milieu plays a role on attractiveness to potential mates has usually been limited to a single source of scent urine [2]. However, most mammals have multiple sources of scent to convey information about sex and receptivity to conspecifics [1,2,10,29]. Studies have shown that different sources of scent also have different sensitivities to steroid hormones [10,29]. In meadow voles, the attractiveness of odors from feces, mouth and the posterolateral region were dependent on testosterone concentration in males and estradiol titer in females. The attractiveness of odors from these sources was eliminated following gonadectomy of the sender. By comparison, the attractiveness of urine and anogenital odors of meadow voles was reduced but not eliminated by gonadectomy [27], suggesting that other hormones, such as prolactin, may mediate the attractiveness of odors to opposite-sex conspecifics [30]. The differences in the sensitivity of substrates to gonadal steroids suggest that each source of scent is sexually distinct and may convey somewhat different information about the attractiveness of that individual to potential mates.

### 1.1. Scent Marks as Indicators of Nutritional Status of Condition of Potential Mates

Although the hormonal milieu of the individual affects scent marking behavior, a large body of literature indicates that the nutritional status, age and identity of an individual also affect the sender and the receiver of scent marks. Two competing hypotheses have been developed to explain the effects of food availability on the sexual behavior of mammals. One hypothesis, the metabolic fuels hypothesis, posits that individuals forgo reproduction when faced with low food availability and invest their limited resources in behaviors not associated with reproduction but with survival [31,32]. Consistent with the metabolic fuels hypothesis are the odor-related behaviors of food-deprived female golden hamsters, musk shrews, *Suncus murinus*, rats, *Rattus norvegicus*, meadow voles and mice [33,34,35,36,37,38]. An alternate hypothesis—the reproduction at all costs hypothesis—states that individuals continue to reproduce or increase reproductive behavior when facing low food availability [39]. For example, Arctic ground squirrels, *Spermophilus parryii* and dasyurid marsupials, *Dasyurus* and *Parantechinus*, reproduce at typical or even higher rates when they are faced with food shortage [39,40]. Both hypotheses consider parental investment tradeoffs between the costs and benefits of delaying or accepting reproduction in the face of food availability [41].

We can modify the reproduction at all costs hypothesis and the metabolic fuels hypothesis to examine the effects of food availability on odor-related behaviors, such as scent marking and self-grooming in mammals [42]. A study on meadow voles yielded two testable hypotheses to determine the effects of food availability on odor-related behaviors [42]. These hypotheses were based on the natural history of meadow voles. First, females may be more likely than males to reduce odor-related behaviors directed at opposite-sex conspecifics when food availability is low and the costs associated with parental investment are high [41,42]. In contrast, males may be less likely than females to forgo mating and scent marking in the face of low food availability or quality, because their reproductive success depends on the number of females they can mate with and they have a low cost of parental investment [41,42]. Data from studies on meadow voles support these two predictions [42].

Food availability also affects the odor-related behaviors of senders and receivers that surround reproduction. Scent-marks provide honest signals of health and nutritional status of senders to receivers [42,43,44]. Thus, the attractiveness of an individual’s scent marks can vary according to the diet and the amount of food a sender consumes [36,45,46,47]. Similarly, the receiver’s nutritional state affects its response to the scent marks of opposite-sex conspecifics [48,49]. However, individuals within a population have unequal access to food [42]. This may be the case even in species with a very specialized diet, given that a particular food source may vary in quantity and quality across an area. For example, if the distribution of food is patchy, dominant individuals are likely to defend a territory that contains more or higher-quality food resources and thus will consume a higher-quality diet than subordinate individuals [50,51]. A higher-quality diet should reflect and be a determinant of that donor’s body condition and social status. In meadow voles, individuals prefer the odors of opposite-sex conspecifics that have been feeding on a high-quality diet compared to those that have been feeding on a lower-quality diet [42]. Female meadow voles that were food deprived for 6 h or longer stopped showing a preference for the odors of a male over those of a female [48]. In addition, food deprivation or restriction of female voles prior to the beginning of postpartum estrus renders their odors and scent marks to be less attractive to males than those of postpartum-estrous female voles that were not food deprived or food restricted prior to the beginning of postpartum estrus [36]. Thus, diet as an adult could affect the attractiveness of an individual’s scent mark to opposite-sex conspecifics.

Likewise, food availability during development could also affect the attractiveness of odors and scent marks produced by individuals when they become adults. Female rat-like hamsters, *Cricetulus triton*, that were food restricted during pregnancy produced male offspring whose odors during adulthood were less attractive to female conspecifics than those of males reared by untreated dams [52,53]. A similar experimental approach has been used in meadow voles, comparing the offspring reared by either untreated dams or dams that were food restricted during early, middle, or late lactation [37,54,55]. Male offspring from dams that were food-restricted during mid lactation produced scent marks that were less attractive to opposite-sex conspecifics than those of male offspring reared by control dams or dams food-restricted during early or late lactation [54,55]. In contrast, female offspring from dams that were food-restricted during early lactation produced odors that were not as attractive as those produced by female offspring from control dams or dams that were food restricted during middle or late lactation [55]. Thus, different sensitive periods for the development of scent-producing tissues may exist between male and female meadow voles [55].

Food availability also affected the amount of time male and female meadow voles spent self-grooming in response to the odors of opposite-sex conspecifics. Self-grooming is a behavior that project the groomer’s odors to nearby conspecifics. Many rodents self-groom when they encounter the scent marks of sexually receptive, opposite-sex conspecifics that are in close proximity [56,57]. By self-grooming individuals make their scents more volatile, allowing them, for a short period of time, to produce odors that are more attractive to opposite-sex conspecifics relative to those of individuals that did not self-groom recently [56,57]. However, male and female meadow voles that were food deprived for either 6 h or 24 h spent less time self-grooming compared to voles that had continuous access to food [58]; these voles are less attractive to opposite-sex conspecifics too [56,57,58].

### 1.2. Scent Marks as Indicators of Age of Potential Mates

Females in many species show a preference for the odors of adult males over those of males around puberty [1,2,59]. For example, female rats are more attracted to the urine of adult males than the urine of prepubescent males [59]. This preference may be associated with the fact that the urine of older male rats contains three compounds, 2-heptanone, 4-methylphenol and 4-ethylphenol, not found in the urine of prepubescent males [59]. Female meadow voles also prefer odors of older males, possibly because such males tend to be heavier and have larger testes [60]. However, as males start to senesce and their health starts to deteriorate, their testosterone titers decrease and the attractiveness of their odors may also decrease [2]. This may occur because a decrease in general condition is reflected in the composition of their odors, or because senesced males cannot invest as many resources into the production of costly sexual signals as younger males. In mice, urine of senescent males contains a lower concentration of major urinary proteins and of the androgen-dependent volatile compounds brevicomin and thiazole compared to middle-aged males [61]. Female mice were less attracted to 12 h old urine of senesced male mice than to that of middle-age male mice, indicating that the reduced content of major urinary proteins in the urine of senesced males affects the longevity and attractiveness of the scent signals [61]. Female mice showed no preference between the fresh scent marks of senesced and middle-aged male mice [61].

The age of the receiver also affects their response to a scent mark. The sensitivity to odors and the ability to discriminate between odors of conspecifics can also decrease in aged animals [62]. Such decrease in olfactory sensitivity can be due to changes in several structures, including the olfactory epithelium, the olfactory bulb and subcortical brain regions and several processes, including loss of selectivity of receptor cells, changes in neurotransmitters and neuronal malfunction due to neurodegenerative disease [62,63]. In a recent study in meadow voles, individuals of up to 13 months of age could distinguish among odors of different conspecifics and they could also distinguish between the odors of males and females [64]. However, 15–18 month-old meadow voles could not differentiate between two individuals of the same sex, although these older voles were able to distinguish between male and female odors, showing a preference for the odors of opposite-sex conspecifics [64]. Thus, aging does not affect the preferences of meadow voles for the odors of sexually receptive, opposite-sex conspecifics over those of same-sex conspecifics. Aging, however, does affects the ability of older voles to discriminate between two different potential mates.

### 1.3. Genotype, Scent Marks and Mate Choice

Scent marks may contain compounds that provide information about the identity of the sender to conspecifics [2,65,66,67]. Thus, scent marks of senders may be sexually and individually distinct [1,2]. I will focus on three compounds of interest: major urinary proteins, Aphrodisin and major histocompatibility complexes. Studies have shown that rodents are able to discriminate between individuals based on these three protein complexes. However, there is limited evidence as to whether differences in these compounds can lead to preferences for particular conspecifics. In male rats and mice, subsets of major urinary proteins (MUPs) provide receivers with information about the identity of sender; this information is essentially a “bar code” of the sender [65,66]. The information in the bar code appears to indicate features of the individual’s genotype and is likely to be stable over time [65,66,67]. In wild male house mice, the subsets of the MUPs and their ratio in the urine were individually distinct [68]. Wild male house mice could discriminate between the subsets of MUPs found in their own urine and those found in the urine of male conspecifics. Male house mice may use this information when competing with other males [68] and females may use this information to distinguish among males [69,70]. A recent study, however, suggests that the MUP profile of a given individual changed over the course of several weeks, supporting more a “dynamic expression” hypothesis than a “barcode” hypothesis [71]. In either case, such information provided by MUPs may be used by house mice to avoid inbreeding and reduce competition with close relatives and also facilitate mate choice.

Information about the sender may also be provided by its production and secretion proteinaceous compounds such as Aphrodisin [72]. Higher concentrations of Aphrodisin are found in the vaginal secretions of female hamsters when they are in estrus. The presence of Aphrodisin in the vaginal secretions of female hamsters induces male hamsters to increase copulatory behavior [73]. Aphrodisin-like proteins were also discovered in the prostate, preputial and salivary glands and liver and uterus in bank voles, *Myodes glareolus* [74]. The urine and saliva of male and female bank voles also contained three odorant-binding proteins, Obp1, Obp2 and Obp3, which may have capacity to bind Aphrodisin-like proteins [74]. Thus, Aphrodisin-like proteins and their binding proteins may be involved in facilitating mate choice [74].

A more defined role in mate choice has been ascribed to the major histocompatibility complexes (MHC). The response of mice to odors of opposite sex conspecifics depends on their respective MHC type [75]. A preference for males that have a different MHC type would allow females to have offspring with a heterozygous MHC type. These offspring would be more likely than those with a homozygous MHC type to respond more effectively to a wider range of pathogens [76,77,78]. In addition, male and female rats and mice can discriminate between the odors of two conspecific females that have a dissimilar MHC [79,80]. Female mice in estrus prefer the odors of males with a dissimilar MHC to those with a MHC that is similar to their own [81]. Such a preference is not maintained if the female mouse was not in estrus and ready to mate [81]. Likewise, peri-ovulatory women prefer men with dissimilar MHC to be more attractive and pleasant compared to that of men that have a MHC similar to their own [82]. Interestingly, women taking oral contraceptives displayed a reduction in the unpleasantness, of men with a similar MHC type to those of men with dissimilar MHC. A sexually receptive female’s preference for males with a MHC type that is different from their own would reduce the likelihood of inbreeding. However, in large groups of captive mice MHC was not as a reliable marker as MUPs in preventing inbreeding [83].

### 1.4. Over-Marking and Mate Choice

I have discussed how different factors such as steroid hormone titers, age, nutritional status and identity can affect the attractiveness of an individual’s scent marks to the opposite-sex conspecifics and its responses to them. The discussion will now shift to the effects of interactions between signalers on the responses of potential mates. I will focus on over-marking. Over-marks are formed when the scent marks of two or more donors overlap [84]. Over-marking occurs in rodents, insectivores, ungulates, carnivores and primates [85,86]. Over-marks provide individuals with the opportunity to directly assess features of two scent donors that may not be available if individuals encountered the scent marks of these two donors separately [87,88]. The top and bottom-scent marks of an over-mark do not mix but remain distinct to provide information about their respective donors. Thus, the top-scent donor and the bottom-scent donor provide information about their association to receivers that can be used as a chemical bulletin board to facilitate mate choice [89,90].

Many studies have examined how rodents perceive and respond to over-marks [85,86,87,88,89,90]. Initial studies on golden hamsters and meadow voles have shown repeatedly that receivers exposed to an over-mark of two donors of the opposite sex later respond preferentially, or have a more selective memory, for the mark of the top-scent donor over that of the bottom-scent donor when the two marks are presented separately [84,89,90]. More recent research, however, suggests that discriminating between and responding to the top-scent and the bottom-scent mark of an over-mark may be more complex than simply preferring or having a selective memory for the top-scent mark [4,91,92]. When the components of the scent mark differ between individuals and interact with the social cues of the over-mark the mate-choice outcome is not always clear. For example, meadow voles were first exposed to same-sex over-marks in which the top and bottom-scent donors differed in gonadal steroid hormone titers. Male voles later preferred the mark of the female donor that had higher titers of estradiol than the female donor that had lower titers of estradiol, independent of which female donor provided the top or the bottom-scent mark in the over-mark. Female voles, however, only expressed a preference for a higher testosterone male if he was also the top-scent donor of a same-sex over-mark [93]. A low testosterone male scent donor that placed a top-mark was equally preferred as a high testosterone bottom-mark donor. Similar results were obtained when meadow voles preferred the top-scent mark if the donor was fed a diet high in protein content but not if top-scent donor was fed a diet low in protein content. Voles preferred the bottom-scent mark if the donor was fed a diet with a higher protein-content [45,94]. Thus, over-marking may be an honest form of signaling that allows receivers to respond positively to the higher quality donor, independent of the position of its scent mark in the over-mark [86].

Ferkin and colleagues have also examined how animals respond to over-marks and the effects of familiarity [95]. In the first experiment, male and female voles were first exposed to an over-mark of two scent donors that were the opposite sex of the subject. Then, the subjects were offered different combinations of the scent marks of the two opposite-sex donors with the scent marks of an opposite-sex, novel scent donor that was not part of an over-mark. The results showed that voles spent more time investigating the scent mark of the top-scent donor to that of the novel, opposite-sex conspecific. In contrast, male and female voles spent similar amounts of time investigating the scent mark of the bottom- scent donor and that of a novel opposite-sex conspecific. The results of the first experiment show that voles were more attracted to the top-scent mark of an over-mark than to that of a scent mark that was not part of an over-mark. Voles, however, were not attracted to the bottom-scent mark over that of a novel scent mark [95]. In the second experiment, voles were exposed repeatedly over four days to an over-mark of the same two opposite-sex donors. Then, as in the first experiment, the subjects were offered the scent marks of the two opposite-sex donors with the scent marks of an opposite-sex, novel scent donor. Sex differences in preferences existed. Male voles spent more time investigating the scent mark of the familiar, top-scent female than the scent mark of a novel female donor [95]. However, male voles spent similar amounts of time investigating the mark of the familiar, bottom-scent female and the mark of a novel female donor [95]. Unlike the responses of male voles, female voles spent more time investigating the mark of a novel male donor than that of either the familiar, top-scent male or that of the familiar, bottom-scent male [95]. These sex differences show that familiarity with scent donors in an over-mark induced different responses by male and female voles to them. Specifically, male and female voles attach different values to the familiar top-scent donor and the familiar bottom-scent donors of an over-mark relative to each other and to a novel scent donor. Females appear to prefer novel males to familiar males and males prefer familiar females only if they occupy the top position in an over-mark. Woodward et al. [91] posited that male voles may view top-scent females as being in possession of that area and the bottom-scent female as being displaced from that area. Female voles that possess territories may be more likely to get pregnant relative to those that do not possess territories [91].

**Figure 1:**
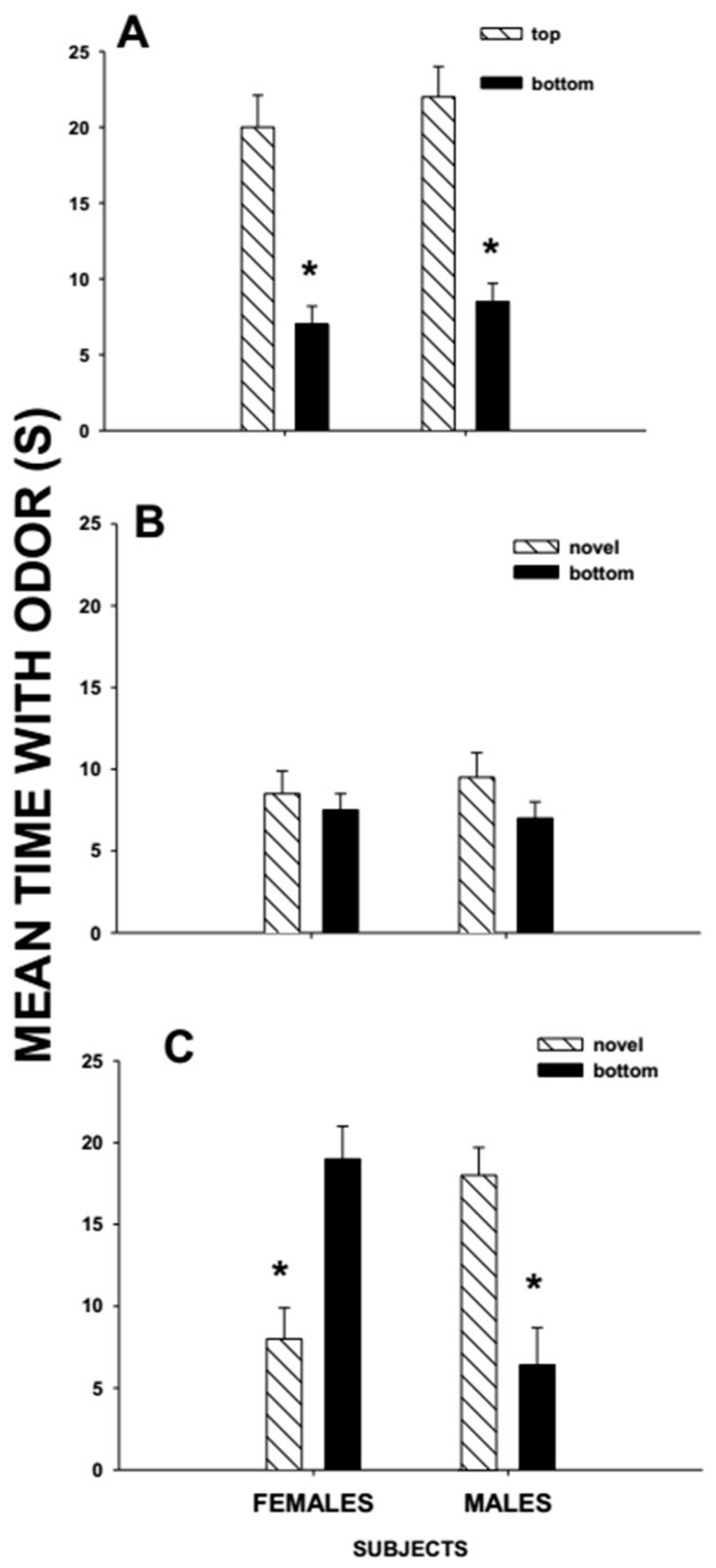
Mean ± SE time (s) that male and female meadow voles investigated the anogenital area scent mark of their (**A**) bottom-scent donor versus that of their top-scent donor; (**B**) novel-scent donor vs. that of their top-scent donor; and (**C**) novel-scent donor vs. their bottom-scent donor in a 3-min preference test after having been exposed to a mixed-sex over-mark in which the top-scent donor was the same sex as the subject and the bottom and novel scent donors were the opposite sex of the subject. * Indicates a significant difference (<0.05) in investigation time between pairs. Based on Woodward et al. [98].

So far, I have described studies in which same-sex conspecifics over-mark the scent marks of conspecifics. However, terrestrial mammals also over-mark the scent marks of opposite-sex conspecifics [86,96,97]. These types of over-lapping scent marks have been characterized as mixed-sex over-marks and studied systematically in meadow voles (Figure 1). Meadow voles exposed to a mixed-sex over-mark, later preferred the scent mark of the opposite-sex donor to that of the same-sex donor, independent of whether the opposite-sex donor was encountered first as the top-scent donor or the bottom-scent mark in the over-mark [98]. Male and female voles showed no preference between the mark of the opposite-sex bottom-scent donor of a mixed-sex over-mark and the mark of an opposite-sex conspecific donor that was not part of the over-mark [98]. Female voles spent more time investigating the mark of the novel male donor than the scent mark of the familiar male conspecific, independent of whether he was the top- or bottom-scent donor of a same-sex over-mark [98]. Thus, female voles may not respond preferentially to a familiar male if his scent mark is part of an over-mark with the scent mark of another female. For males, having one’s scent mark in an over-mark with that of a female may make him less attractive to other female conspecifics [98]. Females that encounter mixed-sex over-marks may view the male scent donor as having just recently mated with that female scent donor, as being sperm depleted. The preference of female meadow voles for a novel male over a familiar male is shared by female house mice [66]. Female voles and mice may prefer novel males to familiar males to encourage sperm competition between males, increase paternity confusion, increase genetic diversity of their offspring by mating with a novel male, or avoid repeated copulations with a familiar partner.

Male voles, in contrast to female voles, treat the scent marks of females that are bottom-scent donors of a mixed sex over-mark as if they are similar to those of novel female donors in their attractiveness [97]. The bottom position of the scent mark of a female in a mixed-sex over-mark may indicate to males that she has association with the top-scent male and may have mated with him. The novel female’s scent mark may signal to the male that she may not be attractive since it was not over-marked by another male [97]. Mating with a female that has already mated or is not attractive may be costly for males in that it increases sperm competition and reduces his likelihood of paternity. Similar conclusions about the role of familiarity and responses to scent marks have been adduced for house mice [68,98] and the white-footed sportive lemur, *Lepilemur leucopus* [99].

In this section, I have discussed studies that have shown that the position of the opposite- sex donor’s mark in the mixed-sex over-mark determines how conspecifics will respond to its scent mark relative to that of a novel, opposite-sex conspecific whose scent mark was not part of an over-mark. In voles, sex differences exist in the responses to mixed sex over-marks. The differences in the responses of males and females to over-marks may represent the costs and benefits of selecting familiar top- or bottom scent donors or novel conspecifics as potential mates [1,2,4,86].

#### The Number of Potential Mates Affects the Responses to Over-Marks

In nature, mammals will enter areas, such as junctions in runways or around a resource where they encounter numerous over-marks. It is likely that in these high-traffic areas, position of the two donor marks in the over-mark may alternate between being the top-scent donor or the bottom-scent donor. Ferkin and co-workers [100] placed male and female meadow voles into an arena that contained a set of either: 10 over-marks and 0 single marks of the conspecific that provided the bottom-scent mark of the over-mark, 9 over-marks and 1 single scent mark of the bottom-scent donor, 8 over-marks and 2 single scent marks of the bottom-scent donor and so on. The results showed that when male voles were placed in an arena containing at least 6 over-marks and 4 single scent marks of the bottom-scent female donor of the over-mark, males spent more time investigating the mark of the top-scent female donor than that of the bottom-scent female donor [100]. When the ratio of over-marks to single scent marks was less than 6:4, the males did not discriminate between the top- and bottom-scent female donors. After female voles entered an arena containing at least 4 over-marks and 6 single marks, they spent more time investigating the mark of the top-scent male donor than that of the bottom-scent male donor. When the ratio of over-marks to single scent marks was less than 4:6, the females did not discriminate between the top- and bottom-scent male donors [100]. Female voles also performed better than male voles in tests that required discriminating between larger and smaller sets of scent marks [101,102,103]. It is also worth pointing out that the proportions of over-marks that male and female voles needed to encounter to display a preference for the mark of the second scent donor over that of the second scent donor were similar to the proportion of scent marks males and females use to over-mark the scent marks of a same-sex conspecific [104]. Thus, male and female voles that are over-marking must be cognizant of the proportion of scent mark of rivals that they over-mark. Individuals that do not over-mark a sufficient proportion of scent marks in an area may be at a disadvantage during mate selection.

### 1.5. Self-Grooming and Mate Choice

Self-grooming may be a form of olfactory communication, akin to scent marking, which allows groomers to transmit information about features of their identity and sex to nearby conspecifics [57,105,106]. Self-grooming may enhance features of this information, which may make nearby conspecifics that encounter these odors more responsive to the groomer [56,57,107]. Several studies provide indirect and direct support for the role of self-grooming in mate attraction. Ground squirrels, deer, *Odocoileus virginianus*, Mongolian gerbils, hedgehogs, *Erinaceus europaeus*, blind mole rats, *Spalax ehrenbergi*, and voles self-groom more when they are exposed to odors of opposite-sex conspecifics than when they are exposed to those of same-sex conspecifics [57]. In another study, male and female meadow voles spent more time self-grooming in response to odors of reproductively active opposite-sex conspecifics than those that were reproductively quiescent [57]. Male meadow voles also spent more time self-grooming in response to odors of a female vole in postpartum estrus than they did in response to odors of females not in postpartum estrus [57]. Male house mice spent more time self-grooming in response to urine of estrus female mice compared to that of female mice not in estrus [108]. Male root voles, *Microtus oeconomus*, spent more time self-grooming in response to urinary odors of lactating females than to urinary odors of non-lactating females [109]. Thus, the sexual condition of the scent donor affects the amount of time that rodents self-groom.

Similarly, gonadectomized male voles, house mice and rats spent less time self-grooming in response to odors of reproductively active opposite-sex females than did their intact counterparts [57,108]. In meadow voles, self-grooming behavior was partially restored by testosterone treatment; whereas self-grooming rates were restored to levels comparable to intact male voles by testosterone therapy [57]. Additionally, food-deprived voles spent less time than those that were not food deprived self-grooming in response to the odors of a sexually receptive, opposite-sex conspecific [109]. Lastly, meadow voles and prairie voles also spent more time self-grooming in response to odors of unfamiliar and unrelated females than in response to those of their reproductively active sisters [110]. In addition, short-photoperiod (nonbreeding season) voles spent similar amounts of time self-grooming in response to the odors of both opposite and same-sex long photoperiod (breeding season) and short-photoperiod scent donors [57]. In contrast, long-photoperiod voles spent more time self-grooming in response to the odors of long photoperiod, opposite-sex conspecifics compared to that in response to the odors of short-photoperiod, opposite-sex conspecifics [57]. Self-grooming is involved in mate selection in voles and likely most rodents.

### 1.6. Sequential Mating and Mate Choice

Male rodents exert mate choice if they are offered simultaneously a mated versus an unmated female conspecific [111,112]. In nature, male rats, mice and voles will be more likely to encounter females sequentially [113,114,115]. Under such conditions, males may or may not be choosy about mating with each female they encounter sequentially [114,116,117,118]. A male should mate with each of the sexually receptive female conspecifics he encounters in non-monogamous species, particularly when parental investment by males is low, the variance in both mating and reproductive success among males is high and the availability of sexually receptive females varies temporally and spatially [41,116]. Theory suggests that males may no longer exert mate choice and mate indiscriminately if it is too costly for them to do so [119,120]. 

Ferkin and Ferkin [121] carried out a study on meadow voles in which they varied the time intervals between successive pairings of females with males to determine if it affected mate choice for a previous mate. The copulatory behavior of male meadow voles was affected by the interval of time between being paired with female 1 and female 2. Specifically, the latency of males to first ejaculation was shorter for the second female than it was for the first female if the interval between pairings was 1 h and was 1 day compared to that for males if the interval between pairings was 4 days, 7 days, or was 10-days [121]. A shorter latency to mate and ejaculate with female 2 than female 1 may be attributed to heightened sexual arousal by the male. Meadow voles, like many male mammals [122], which mate with multiple females [123], are susceptible to the Coolidge Effect. The Coolidge Effect occurs when a previously sexually-sated male will exhibit arousal and high sexual performance given the introduction of a new, sexually receptive female [122]. For male meadow voles it seems that the Coolidge Effect has a relatively short time frame, somewhere around 24 h.

In addition, when the interval between pairings was 1 h the time between first and last ejaculation was shorter for female 2 than it was for female 1 [121]. A shorter interval between first and last ejaculation would shorten the length of the copulatory bout [124,125]. This would provide males with more time to locate and visit other females, potentially increasing mating success. A shorter copulation bout may also provide benefits to female voles. Females may have more time for evaluating additional, potential suitors while facilitating sperm competition between sires [126,127]. A decrease in the length of a copulatory bout with female 2 was not observed in males tested with pairing intervals of 4 days, 7 days, or 10 days. This finding suggests that male meadow voles may view intervals of at least 4 days between successive pairings with two different females as being temporally disconnected and not sequentially-distinct events.

Ferkin and Ferkin [121] found that each of the male voles they tested attempted to mate with the two females they encountered sequentially. In that approximately 1 in 4 males in that study failed to copulate with either female 1 or female 2, it appears that female voles exert mate choice. Thus, female choice may contribute to the reported high variance in mating success and reproductive success of male meadow voles [126,128]. This variance coupled with differences among male voles in locating a willing, sexually receptive female [129] may induce meadow voles to mate indiscriminately with each female he encounters. Male meadow vole may benefit from employing such a strategy, especially if the benefits of doing so outweigh their costs.

Male meadow voles can detect changes in the odors of two previous mates depending on how far along each female was in gestation. Male voles do not distinguish between two female mates unless 4–6 days have separated the male’s mating bout with each female [121]. It may take about a week for a male meadow vole to forget or place a lower value on the scent mark of a previous mate compared to that of a more recent mate [130]. This may allow male meadow voles to choose this female as a mate at a later time. Second, male voles treated the odors of female 1 and female 2 as being different in their attractiveness when the interval between pairings was 7 and 10 days. Perhaps male voles detected some change in the odor of a previous mate 7 and 10 days after mating. In voles, implantation of the blastocysts into the uterine wall occurs within 5–6 days of mating [131,132]. Male meadow voles may spend more time investigating the odor of female 2 to that of female 1 on days 7 and 10 because female 2 had not yet implanted her blastocysts whereas female 1 had her blastocysts implanted already. Male voles did not discriminate between his previous mates when the interval was 4 days or less, which is shorter than the time it takes for blastocysts to implant. A male meadow vole may be able to detect changes in the odor of a female once she has implanted the blastocyst around day 5 or 6 of gestation. Stud male prairie voles continue to visit or interact with a female after mating [133,134]. Presumably, a male does so to prevent pregnancy disruption and to increase the likelihood that the eggs he fertilized become implanted into the female’s uterine wall [135,136]. Thus, male meadow voles may no longer need to discriminate between female 1 and female 2 if the mating interval was 14 days and both females were past blastocyst implantation.

### 1.7. Mate Choice and Public Information

Male voles use social information provided by the scent marks of both rival males and that of a female conspecific to influence their odor preferences for that female [2,137,138]. Specifically, male voles exposed first to the bedding of females that had previously associated with the bedding of 3 or 5 males had a shorter latency to mate and had a shorter copulatory bout compared to that of males that mated with females previously associated with the bedding of 0 males, 1 male, or 2 males [137]. Similarly, male house mice and rats will decrease the time spent in copulating as the risk and intensity of sperm competition increase [124,139,140]. Male meadow voles may have to reach some threshold number of suitors associated with a female, in this case 3, before they modify their copulatory behavior. Male voles may view a female vole that may have been visited by at least 3 males as being more fertile, less stringent in assessing a male’s condition or quality, and/ or more willing to mate compared to females with fewer suitors [137]. The preference for the “popular” female may facilitate mate copying [103,137,141,142,143].

Individuals can gain social information and identify potential mates by observing the choices of conspecifics [2,137,142,143]. For example, female Norway rats spent more time in close proximity with a male that had recently engaged in coitus compared to a male that had not recently engaged in coitus. Female rats displayed such a preference for the mated male even when they did not observe that male’s mating bout [142]. In addition, female rats in estrus copulated more often with males that had recently mated with another female compared to males that had not recently mated with a female. Moreover, male rats that mated recently mounted the focal female more frequently and ejaculated sooner than did males that had not recently copulated. Galef et al. [142] also discovered that female rats found the odors of male rats that had recently copulated to be more attractive than were the odors of males that had not recently copulated. Female mice displayed a preference for the odors of males that were associated with the odors of a conspecific female in estrus and that this preference lasted 24 h [143]. In addition, female mice could discriminate between males subclinically infected with the gastrointestinal nematode parasite, *Heligimosomoides polygyrus* and nonparasitized male conspecifics. Females avoided the odors of parasitized male mice. Females no longer avoided and preferred the odors of parasitized males if they were associated with the odors of a female mouse in estrus [143]. Additional work showed that oxytocin mediated the ability of female mice to respond to differences in the odors of nonparasitized males and parasitized males [143]. These findings suggest that OT genes are necessary for the processing of inadvertent social information and likely the integration of both direct and indirect social information [143].

Using this public information, however, may be costly for males [144]. A male that mates with a female visited by 3 or more males would lower his paternity assurance, whereas mating with the female that had zero or one mate would increase his paternity assurance and indirect benefits [145]. The presence of scent marks can also affect sperm competition. When another male’s odors were present, male voles increased the number of sperm that they delivered to their mate [127]. When a male vole mates while being exposed to the odors of 5 other males, the amount of sperm in his ejaculate is intermediate between that if the odor of one male was present and the amount of sperm in his ejaculate if no odors of other males were present [146]. When a male meadow vole mates with a female in the presence of odors from a food-deprived male, he does not increase the amount of sperm in his ejaculate [147]. Thus, male voles assign a lower competitive threat to odors of food-deprived males than to odors of ad-libitum fed males [2]. For male meadow voles the number and relative quality of the male scent donors affects the amount of sperm in his ejaculate; it does not affect his willingness to mate with that female [136,146,147]. Male house mice, however, do not increase the number of sperm in their ejaculates in response to a high level of sperm competition conveyed by odors of other males [148]; male mice instead are less willing to mate with that female [140].

## 2. Conclusions

The scent marks deposited by the sender must first be detected by potential mates to facilitate mate choice [2,138]. Because scent marks are typically deposited on prominent objects or along paths that are shared with conspecifics they are likely to be detected by conspecifics. Mate choice depends on detecting the cues of conspecifics and involves cognitive processes for acquiring, processing, retaining and acting on social information [129]. The scent marks would provide public information to individuals that encounter them and in essence be a chemical bulletin board [89,90]. This chemical bulletin board would provide information that may be directed towards specific receivers such as potential mates. Alternatively, the information can be viewed by non-targeted individuals such as rivals and heterospecifics; in this case the information would be inadvertent and public [2,143]. How such information will be treated depends on its value to the receiver [2], phenotype and genotype of the sender and receiver [68,69,149,150,151,152,153,154] and the social context. However, ecological challenges such as the availability of mates and resources can cause variation in life history strategies and characteristics associated with communicating with the opposite sex and reproducing [155,156]. Under such conditions, the decision and ability to attract mates and respond to the signals of potential mates will depend on the life history constraints placed on individuals such as their fecundity, sex, lifespan, opportunities to mate in the future and age at senescence [41,42,138,155,156].
